# Emergency medical services utilization in acute stroke in Qatar - an observational cohort study

**DOI:** 10.1186/s12245-025-00877-5

**Published:** 2025-03-31

**Authors:** Zain A. Bhutta, Naveed Akhtar, Tim Harris, Maaret Castren, Yahia Imam, Sameer A. Pathan, Guillaume Alinier, Saadat Kamran, Peter A. Cameron, Tuukka Puolakka

**Affiliations:** 1 Department of Emergency Medicine, Hamad Medical Corporation, Doha, Qatar; 2 Department of Emergency Medicine and Services, Helsinki University Hospital and University of Helsinki, Helsinki, Finland; 3 Department of Neurology, Neuroscience Institute, Hamad Medical Corporation, Doha, Qatar; 4 Blizard Institute of Barts & The London School of Medicine, Queen Mary University of London, London, UK; 5 Royal Adelaide Hospital, Adelaide, South Australia; 6 School of Public Health and Preventive Medicine, Monash University, Melbourne, Australia; 7 Hamad Medical Corporation Ambulance Service, Doha, Qatar; 8 Weill Cornell Medicine-Qatar, Education City, Doha, Qatar; 9 School of Health and Social Work, University of Hertfordshire, College Lane, Hatfield, UK; 10 Faculty of Health and Life Sciences, North Umbria University, Coach Lane Campus, Newcastle upon Tyne, UK; 11 The Alfred Hospital, Emergency and Trauma Centre & School of Public Health and Preventive Medicine, Monash University, Melbourne, Australia; 12 MedSTAR Emergency Medical Retrieval Service, South Australia Ambulance Service, Adelaide, Australia

**Keywords:** Emergency medical services, Stroke, EMS utilization, Qatar

## Abstract

**Introduction:**

Timely recanalization improves long-term outcomes in acute ischemic stroke (IS) patients, but most patients present outside the therapeutic window. Emergency Medical Services (EMS) can reduce pre-hospital delay and increase the likelihood of recanalization. We aim to determine the characteristic variations amongst suspected acute stroke patients using EMS.

**Methods:**

This retrospective observational study included all suspected acute stroke patients admitted to a national tertiary care hospital in Qatar from January 2014 to September 2020. We evaluated demographics, clinical features, treatment impact, and associated factors in EMS versus non-EMS transported groups.

**Results:**

During the study period, 11,892 patients presented with suspected stroke. Of these, 65.1% used EMS (EMS group) for transportation to the hospital. Median age was comparable between EMS and non-EMS group [52 years; IQR 43–63 vs. 43–62, *p* < 0.05]. Male to female ratio was 3:1. EMS use in the Qatari population (59.2%) was relatively low. Patients with hemorrhagic stroke (82.4%) had significantly higher EMS use as compared to IS (65.7%) and cerebral venous thrombosis (64.7%); *p* < 0.001. Symptom onset to ED presentation time was lower in EMS users, with 41.0% arriving within 4.5 h vs. 24.3% in the non-EMS transported group (*p* < 0.05). Patients with unilateral weakness (66.4%), aphasia (78.2%), neglect (78.2%), dysarthria (68.4%), loss of consciousness (83.3%), and seizures (83.9%) were more likely to use EMS than alternative modes of transportation. Patients attending via EMS had higher rates of thrombolysis than others (82.4% vs. 17.6%; *p* < 0.001) and a shorter door-to-needle time (56.4 *±* 38.2 min vs. 75.7 *±* 43.8 min; *p* < 0.001).

**Conclusion:**

EMS utilization in acute stroke patients was high and was associated with rapid and higher rates of therapeutic intervention. However, younger age, Arab ethnicity, and less obvious stroke symptoms were associated with lower EMS use, emphasizing the need for targeted public health interventions to improve EMS activations.

**Supplementary Information:**

The online version contains supplementary material available at 10.1186/s12245-025-00877-5.

## Introduction

Stroke, a leading cause of morbidity and mortality worldwide, demands timely and specialized medical treatment for optimal patient outcomes. Approximately 70–80% of strokes in Qatar are ischemic [1, 2]. Timely reperfusion therapy in ischemic stroke (IS) patients reduces the chances of disability as compared to patients who do not receive timely treatment [3, 4]. However, the majority of IS patients are not eligible for such treatment due to contraindications or late presentation, with around 3.4–7.3% of all acute presentations being eligible [5–9]. In Qatar, the proportion of patients admitted to the hospital within 3 h of stroke onset is 18% [2]. Previous studies have identified pre-hospital delay as one of the major factors contributing to the delay in effective stroke management and poor outcomes [5, 10, 11]. Prompt identification of the initial signs and symptoms of stroke and early transportation via Emergency Medical Services (EMS) is key to decreasing the prehospital times and increasing the probability of IS patients receiving reperfusion therapy.

A study conducted in the United States estimated that if the stroke onset time was known and EMS were immediately activated at stroke onset, the proportion of patients receiving tPA treatment would increase from 4.3–28.6% [12]. In efforts to increase early recognition of stroke and rapid transfer to hospital, public health interventions such as the ‘Act FAST’ (Face, Arm, Speech and Time) campaign have focused on public awareness of stroke signs and symptoms and prompt activation of EMS at stroke onset. Such campaigns have been carried out globally with varying results and success rates [13, 14, 15]. A similar campaign conducted in Qatar in 2015 focused mainly on disseminating knowledge and increasing public awareness of stroke signs and symptoms, and the need for rapid hospital attendance by activating EMS. A study analyzing the impact of this stroke awareness campaign in Qatar reported that 20.1% of the participants were aware of the signs and symptoms of stroke, while 33.3% of all participants activated EMS at stroke onset [16].

Previous studies in Qatar suggest significant efforts are required to increase stroke awareness and encourage the use of EMS [17, 18]. The aim of this study was to determine the characteristics and demographic variation amongst the patients using EMS at stroke onset and its association with stroke management and outcomes in Qatar.

## Methods

This retrospective, observational cohort study analyzed all patients admitted to the stroke service at the national tertiary care hospital in Qatar between January 2014 and September 2020. All patients with stroke were prospectively enrolled in the National Stroke Registry [19]. The study was conducted according to the ethical principles outlined in the 1975 Declaration of Helsinki and was approved by the Institutional Review Board (IRB) for Human Subject Research (HSR) at Hamad Medical Corporation (HMC) (MRC-01-20-1135).

### Study setting

Qatar’s population of approximately 3 million is characterized by significant ethnic diversity, with expatriates constituting around 88% and males accounting for nearly 75% of this population. The local Qatari population constitutes a small portion of this demographic (12%), while major expatriate groups include South-Asians, Non-Qatari Arabs, and people from the Far-East [19, 20]. Hamad Medical Corporation, the only government tertiary healthcare provider in Qatar, provides admission and stroke care to all acute stroke patients in the country; private hospitals do not provide care for patients with acute stroke [17]. Stroke services at HMC hospitals have been previously described [21].

HMC operates the National Emergency Medical Services (EMS) provider in Qatar, the Hamad Medical Corporation Ambulance Service (HMCAS). The HMCAS operates with a workforce of approximately 1300 clinical and support personnel, along with a fleet of over 200 ambulances, 22 rapid response vehicles, and three helicopters [22]. To ensure prompt response to emergency calls, HMCAS utilizes a “hub and spoke” deployment model [23].

### Data

Data were collected prospectively as patients presented to the Emergency Department with suspected strokes and were subsequently validated retrospectively using patient medical records by a dedicated stroke research team as part of the National Stroke Registry. The data consisted of demographic variables including sex, age, and nationality [further categorized into ethnic groups as Arabs, Asians (including South Asians and Far Eastern populations), Africans, and Caucasians]. Stroke-related variables included stroke type [categorized as Ischemic stroke (IS), transient ischemic attack (TIA), intracranial hemorrhage (ICH), cerebral venous sinus thrombosis (CVST), and stroke mimics], stroke severity, and presentation. The National Institute of Health Stroke Scale (NIHSS) score at initial assessment and discharge, risk factors, door-to-needle time (DNT), thrombolysis, thrombectomy, duration of stay, modified Rankin Score (mRS) at discharge, mortality, and any inpatient complications were recorded. The “Trial of ORG 10172” in Acute Stroke Treatment (TOAST) criteria was used to classify IS [24]. Modes of patient arrival was divided into two groups: those who arrived at the hospital via EMS and those who arrived via any other means (such as taxis, buses, or private vehicles). The primary outcome was the use of EMS, defined as the proportion of patients who called 999 (Qatar Emergency Services contact number) and utilized HMCAS as the mode of transportation to the hospital at stroke onset.

### Statistical analysis

All categorical variables were reported as frequencies and corresponding percentages. All continuous variables were reported as mean with standard deviation for parametric data and as medians with inter-quartile range for non-parametric data. The normality of the continuous variables was assessed using the Shapiro-Wilk test. For comparative analysis, patients who used EMS were compared to patients who did not use EMS, with categorical variables analyzed using the chi-square test and continuous variables using the Wilcoxon test. Additionally, odds ratio (OR) with 95% confidence interval were reported for all variables to assess the strength and precision of associations. Logistic regression analysis was utilized to evaluate factors associated with EMS utilization among stroke patients. A stepwise approach was applied to construct the logistic regression model, employing the backward elimination technique. Univariate analyses were performed on all variables that could potentially influence the outcomes. Variables with unadjusted *p*-values less than 0.2 were selected for inclusion in the multivariable model. At each iteration, the variable with the highest *p*-value was removed, and its potential confounding effects on the remaining variables were assessed. If no confounding was detected and the variable remained non-significant, it was excluded from the model. The level of significance for the multivariable analysis was defined as a *p*-value of 0.05. All statistical analysis was performed using Stata (version 14 MP, StataCorp, 58 College Station, USA).

## Results

During the study period, 11,892 patients were admitted to the stroke service, with 75.3% (*n* = 8,959) males. The median age of the cohort was 52 (42–62) years. Of all the stroke admissions, 7,734 (65%) of the patients utilized EMS for transportation to the hospital. Among the suspected stroke patients who utilized EMS for transportation to the hospital, 73.2% were confirmed to have a stroke diagnosis. This included 49.4% (*n* = 3,820) with IS, 8.7% (*n* = 675) with TIA, 13.8% (*n* = 1,072) with ICH, and 1.3% (*n* = 99) with CVST. Patients in the EMS group were more likely to be male (OR, 1.21; 95% CI, 1.11–1.32; *p* < 0.001), non-Arabs (specifically Asians, including those from South Asian and Far Eastern background) (OR, 1.23; 95% CI, 1.14–1.33; *p* < 0.001), have a diagnosis of ICH (OR, 2.76; 95% CI, 2.37–3.21; *p* < 0.001), have hypertension (*p* < 0.001), atrial fibrillation (AF)(*p* < 0.001) and/or chronic kidney disease (CKD)(*p* < 0.05), and a higher NIHSS at admission (*p* < 0.001) (Supplementary Table 1). Patients in the EMS group also had a higher likelihood of having large vessel disease or cardioembolic stroke, as determined by the TOAST criteria for IS (Table 1).

Intravenous thrombolysis was administered to 12.4% (*n* = 725) of IS patients during the study period. In comparison to the non-EMS group (6.1%), IS patients in the EMS group (15.8%) were significantly more likely to receive intravenous (IV) tPA (OR, 2.87; 95% CI, 2.33–3.54; *p* < 0.001) (Table 2). Thrombectomy was performed in 4.6% of the patients, with a higher likelihood of undergoing the procedure among EMS users (6%) compared to those in the non-EMS group (1.9%) (*p* < 0.001). The median door-to-needle time in the EMS group was 49 (31–69) minutes, which was substantially lower than in the non-EMS group [62 (47–99) minutes (*p* < 0.001)] (Table 3). In the non-EMS group, a greater proportion of patients (3.1% vs. 2.4%; *p* < 0.05) reported a stroke onset time of less than one hour than in the EMS group. However, the EMS group exhibited a substantially higher proportion of patients who reported a stroke onset time of 1–4.5 h (38.6% vs. 21.3%; *p* < 0.001) and wake-up strokes (2.7% vs. 1.7%; *p* < 0.001) (Supplementary Table 3). A total of 67.9% (*n* = 8,064) of patients achieved a favorable outcome at discharge, defined by an mRS of 0–2. Patients with TIA (93.9%), CVST (83.6%), and stroke mimics (85.1%) had significantly better outcomes at discharge compared to those with IS (60.2%) and ICH (31.8%).

**Fig. 1:**
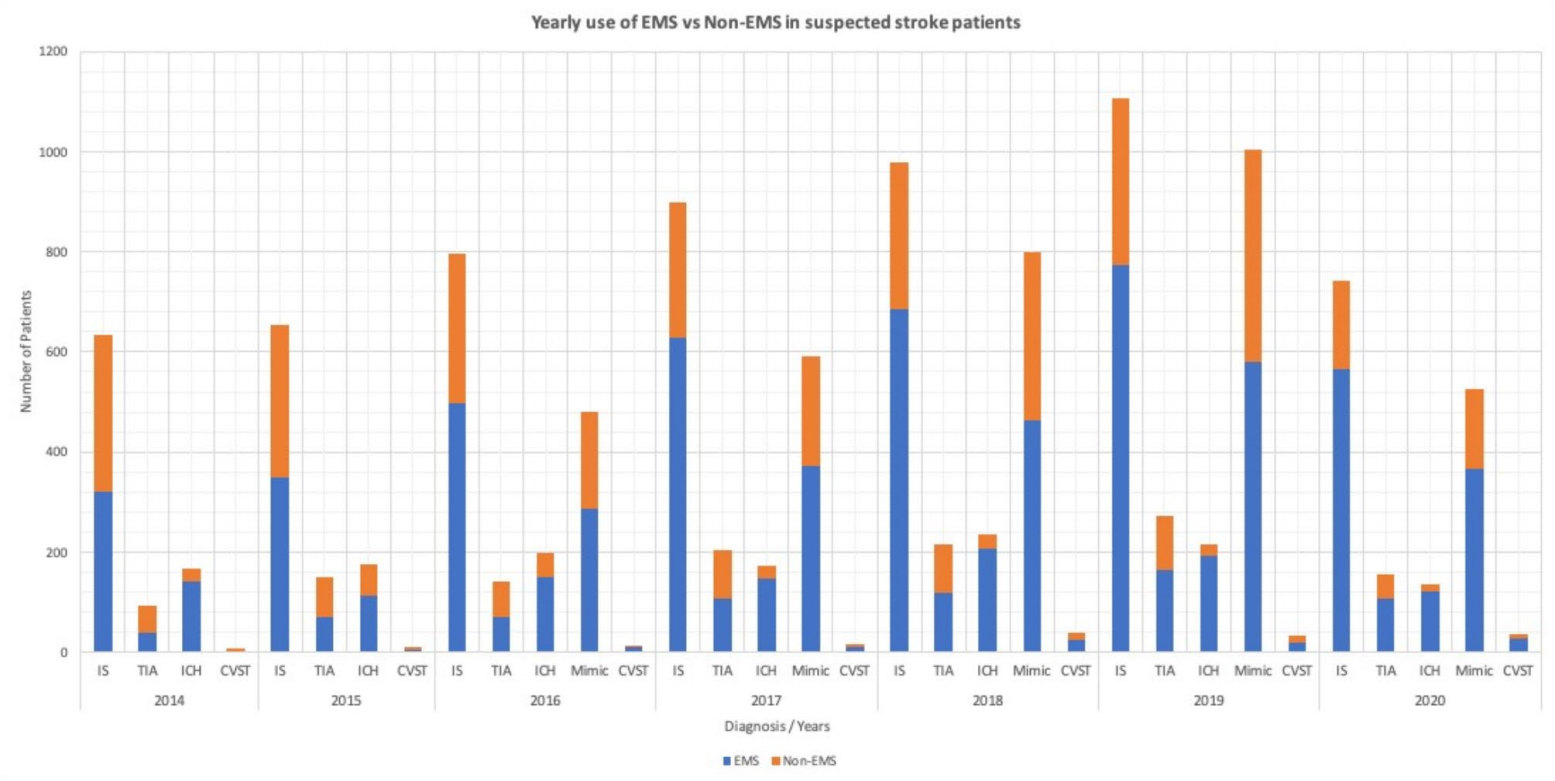
Yearly trends in EMS and non-EMS transportation for suspected stroke patients during the study period The EMS (Emergency Medical Services) group is defined as patients who utilize ambulance services as a mode of transportation to the hospital at stroke onset, while the non-EMS group is defined as patients who utilize any other mode of transportation. IS: Ischemic stroke, TIA: Transient Ischemic Attack, ICH: Intracerebral Hemorrhage, CVST: Cerebral venous sinus thrombosis

The data indicates an overall rising trend in EMS utilization among stroke patients from 2014 (6.5%) to 2020 (15.4%), with an average annual growth rate of approximately 33.3%. In 2014 and 2015, 55.6% and 53.8% of all stroke patients, respectively, opted for EMS as their mode of transportation, rising to 62.2% in 2016, 67.4% in 2017, 66.1% in 2018, 65.8% in 2019, and reaching 74.5% in 2020 (Fig. 1). Additionally, an increasing trend of stroke mimics was observed among the suspected stroke patients, increasing from 29.5% in 2016 to 38.2% in 2019 before declining to 33% in 2020, while EMS utilization among stroke mimics fluctuated over the years, with a decrease from 59.6% in 2016 to 57.8% in 2018, followed by a significant increase to 69.4% in 2020 (Supplementary Table 2). Considering the potential impact of the COVID-19 pandemic on healthcare seeking behaviors, a comparative analysis assessing differences between pre-2020 (2014–2019) and 2020 data revealed a significant increase in EMS utilization during 2020 (74.5% vs. 63.5%; *p* < 0.001). However, no significant differences were observed in median age (52 vs. 52 years, *p* = 0.06), sex distribution (73.5% vs. 75.6% male, *p* = 0.07), or NIHSS at admission (2 vs. 2, *p* = 0.16). Thrombolysis (7.7% vs. 13.2%; *p* < 0.001) and thrombectomy (2.3% vs. 4.9%; *p* < 0.01) rates were significantly lower in 2020, and door-to-needle times were longer (median: 62.5 min vs. 50 min, *p* = 0.051). Despite these differences, survival at discharge was slightly higher in 2020 (98.4% vs. 97.5%; *p* < 0.05) (Supplementary Table 4).

**Table 1:** Characteristics and risk factors of suspected stroke patients using EMS vs. non-EMS as mode of transportation to the hospital

Variables	Category	Use of Emergency Medical Services (EMS), *n* (%)		Total	Odds Ratio	95% CI	*p*-value
		Yes, *n* = 7734 (%)	No, *n* = 4158 (%)	*N* = 11,892 (%)			
Age (Median, IQR*) (years)*		52 (43–63)	52 (43–62)	52 (43–62)			< 0.05
Age categorization (n, %) (years)	Below 24	63 (0.8)	51 (1.2)	114 (0.9)	0.66	0.45–0.97	< 0.05
	24–44	2143 (27.7)	1149 (27.6)	3292 (27.7)	1.00	0.92–1.09	0.92
	44–65	4001 (51.7)	2217 (53.3)	6218 (52.3)	0.93	0.86–1.01	0.10
	65–80	1222 (15.8)	612 (14.7)	1834 (15.4)	1.08	0.97–1.21	0.11
	80 and above	303 (3.9)	129 (3.1)	432 (3.6)	1.27	1.02–1.58	0.023
Sex	Male	5925 (76.6)	3034 (72.9)	8959 (75.3)	1.21	1.11–1.32	< 0.001
Population	**Arabs**	2653 (34.3)	1628 (39.2)	4281 (35.9)	0.81	0.75–0.87	< 0.001
Subgroup	Qatari	1323 (17.1)	913 (21.9)	2236 (18.8)	0.73	0.66–0.80	< 0.001
	**Non-Arabs**	5081 (65.7)	2530 (60.8)	7611 (64.0)	1.23	1.14–1.33	< 0.001
Subgroup	Asian (SA + FE)	4508 (58.3)	2245 (53.9)	6753 (56.8)	1.19	1.10–1.28	< 0.001
	African	358 (4.6)	178 (4.3)	536 (4.5)	1.08	0.89–1.31	0.38
	Caucasian	205 (2.6)	103 (2.5)	308 (2.6)	1.07	0.84–1.37	0.57
	Other	10 (0.1)	4 (0.09)	14 (0.1)	1.34	0.38–5.87	0.61
Diagnosis	Ischemic stroke	3820 (49.4)	1990 (47.8)	5810 (48.6)	1.06	0.98–1.14	0.11
	TIA	675 (8.7)	551 (13.3)	1226 (10.3)	0.62	0.55–0.70	< 0.001
	ICH	1072 (13.8)	229 (5.5)	1301 (10.9)	2.76	2.37–3.21	< 0.001
	Mimic	2068 (26.7)	1334 (32.1)	3402 (28.6)	0.77	0.71–0.84	< 0.001
	CVST	99 (1.3)	54 (1.3)	153 (1.3)	0.98	0.69–1.40	0.93
BMI (Median, IQR)		27.3 (24.4–30.6)	27.3 (24.4–30.8)	27.3 (24.4–30.7)			0.22
HBA1C (Median, IQR)		6.1 (5.5–8.1)	6.2 (5.5–8.3)	6.2 (5.5–8.2)			0.13
Comorbids	Diabetes	3686 (47.7)	2009(48.3)	5695 (47.8)	0.97	0.90–1.05	0.49
	Hypertension	5321 (68.8)	2630 (63.2)	7951 (66.8)	1.28	1.18–1.38	< 0.001
	Dyslipidemia	3523 (45.6)	1939 (46.6)	5462 (45.9)	0.95	0.88–1.03	0.25
	DVT	24 (0.3)	14 (0.3)	38 (0.3)	0.92	0.45–1.92	0.80
	CAD	820 (10.6)	423 (10.2)	1243 (10.4)	1.04	0.92–1.18	0.46
	AF	512 (6.6)	199 (4.8)	711 (5.9)	1.41	1.18–1.67	< 0.001
	CHF	20 (0.3)	9 (0.2)	29 (0.2)	1.19	0.52–2.98	0.65
	CKD	300 (3.9)	131 (3.2)	431 (3.6)	1.24	1.00-1.54	< 0.05
Prior stroke		915 (11.8)	482 (11.6)	1397 (11.7)	1.02	0.91–1.15	0.69
Prior TIA		66 (0.9)	47 (1.1)	113 (0.9)	0.75	0.51–1.12	0.13
TOAST criteria for AS (*n* = 5802, % of IS for each category)	Small vessel disease	1691 (44.3)	1117 (56.2)	2808 (48.4)	0.62	0.55–0.69	< 0.001
	Large vessel disease	902 (23.6)	350 (17.6)	1252 (21.6)	1.44	1.26–1.66	< 0.001
	Cardioembolic	716 (18.7)	290 (14.5)	1006 (17.3)	1.35	1.16–1.57	< 0.001
	Stroke of determined origin	381 (9.9)	184 (9.2)	565 (9.7)	1.08	0.90–1.31	0.37
	Stroke of undetermined origin	124 (3.2)	47 (2.3)	171 (2.9)	1.38	0.97–1.99	0.058

EMS (Emergency Medical Services) group is defined as patients who utilized ambulance services as mode of transportation to the hospital at stroke onset, while non-EMS group is defined as patients who utilized any other mode of transportation. IQR: Interquartile range, CI: Confidence Interval, TIA: Transient Ischemic Attack, ICH: Intracerebral Hemorrhage, CVST: Cerebral venous sinus thrombosis, BMI: Body Mass Index, DVT: Deep vein thrombosis, CAD: Coronary artery disease, AF: Atrial fibrillation, CHF: Congestive Heart Failure, CKD: Chronic Kidney Disease, HbA1C: Glycated hemoglobin and TOAST criteria: Trial of Org 10,172 in acute stroke treatment criteria

**Table 2:** A comparison of the hospital course, management and outcomes of suspected stroke patients using emergency medical services vs. non-emergency medical services users

Variables	Category	Use of Emergency Medical Services (EMS), *n* (%)		Total	Odds Ratio	95% CI	*p*-value
		Yes, *n* = 7734 (%)	No, *n* = 4158 (%)	*N* = 11,892 (%)			
NIHSS^a^ at admission, n I (%)	0 (No symptom)	2069 (27.3)	1536 (37.4)	3605 (30.8)	0.63	0 0.58–0.68	< 0.001
	1–4	2846 (37.5)	1847 (44.9)	4693 (40.1)	0.73	0.68–0.79	< 0.001
	5–15	1904 (25.1)	586 (14.3)	2490 (21.3)	2.01	1.82–2.23	< 0.001
	16–20	374 (4.9)	67 (1.6)	441 (3.8)	3.13	2.41–4.06	< 0.001
	20 and over>	397 (5.2)	74 (1.8)	471 (4.0)	3.01	2.34–3.86	< 0.001
Door to needle time (Median, IQR), mins		49 (31–69)	62 (47–99)	52 (34–73)			< 0.001
Thrombolysis (tPA given) n, % of IS	Total	645 (16.8)	138 (6.9)	783 (13.4)	2.65	2.18–3.21	< 0.001
	Ischemic stroke	603 (15.8)	122 (6.1)	725 (12.4)	2.87	2.33–3.54	< 0.001
	TIA	4 (0.1)	5 (0.2)	9 (0.1)	0.65	0.12–3.04	0.52
	Mimic	37 (0.9)	11 (0.5)	48 (0.8)	2.19	1.08–4.77	< 0.05
	CVST	1 (0.02)	0 (0)	1 (0.02)			0.45
Thrombectomy, n (% of IS)		230 (6.0)	37 (1.9)	267 (4.6)	3.4	2.4–4.8	< 0.001
Disposition, n (%)	Home	5139 (66.5)	3367 (80.9)	8506 (71.5)	0.46	0.42–0.51	< 0.001
	Rehab	1324 (17.1)	320 (7.7)	1644 (13.8)	2.47	2.17–2.81	< 0.001
	Long term care	285 (3.7)	68 (1.6)	353 (2.9)	2.30	1.76-3.00	< 0.001
	Died in Hospital	219 (2.8)	66 (1.6)	285 (2.4)	1.80	1.36–2.38	< 0.001
	Other specialty	767 (9.9)	337 (8.1)	1104 (9.3)	1.24	1.09–1.42	< 0.01
Length of stay (mean *±* SD) in days	Total population	5.7 *±* 8.7	4.3 *±* 9.4	5.2 *±* 9.0			< 0.001
	Ischemic stroke	6.4 *±* 9.5	5.05 *±* 5.9	5.9 *±* 8.5			< 0.001
	TIA	2.08 *±* 2.1	2.6 *±* 8.7	2.3 *±* 6.1			0.42
	ICH	11.7 *±* 11.3	11.2 *±* 17.0	11.6 *±* 12.4			< 0.01
	Mimic	2.4 *±* 3.1	2.6 *±* 11.4	2.5 *±* 7.5			< 0.001
	CVST	8.5 *±* 8.4	5.9 *±* 4.9	7.6 *±* 7.4			0.08
Discharge mRS^b^ 0–2, n (%)	Total	4774 (61.9)	3290 (79.4)	8064 (67.9)	0.43	0.39–0.46	< 0.001
	Ischemic stroke	2080 (54.5)	1409 (71.1)	3489 (60.2)	0.72	0.66–0.78	< 0.001
	TIA	622 (92.2)	529 (96.2)	1151 (93.9)	0.59	0.53–0.68	< 0.001
	ICH	303 (28.3)	111 (48.5)	414 (31.8)	1.48	1.19–1.85	< 0.001
	Mimic	1687 (81.9)	1195 (89.9)	2882 (85.1)	0.69	0.63–0.75	< 0.001
	CVST	82 (82.8)	46 (85.2)	128 (83.6)	0.96	0.67–1.37	0.82
mRS^b^ 0–2 at 90 days follow up, n (%) (*n* = 7005)	Total	3032 (66.6)	2064 (84.0)	5096 (72.7)	0.65	0.61–0.71	< 0.001
	Ischemic stroke	1753 (63.9)	1160 (80.0)	2913 (41.6)	0.75	0.69–0.83	< 0.001
	TIA	533 (93.2)	482 (96.7)	1015 (14.5)	0.56	0.49–0.64	< 0.001
	ICH	304 (39.8)	97 (56.4)	401 (5.7)	1.71	1.36–2.16	< 0.001
	Mimic	377 (94.5)	283 (96.6)	660 (9.4)	0.70	0.56–0.82	< 0.001
	CVST	65 (90.3)	42 (95.4)	107 (1.5)	0.83	0.56–1.22	0.35
Dead at discharge (n, % of deaths for each category)	Total	211 (2.7)	66 (1.6)	277 (2.3)	1.74	1.32–2.29	< 0.001
	Ischemic stroke	80 (2.1)	35 (1.7)	115 (0.9)	1.19	0.80–1.77	0.38
	TIA	0	0	0			
	ICH	115 (10.7)	27 (11.8)	142 (1.2)	0.89	0.57–1.39	0.63
	Mimic	16 (0.8)	3 (0.2)	19 (0.2)	3.45	1.07–11.1	< 0.05
	CVST	0	1 (1.8)	1 (0.01)			
Complications, n (%)	Pneumonia	375 (4.8)	79 (1.9)	454 (3.8)	2.63	2.05–3.40	< 0.001
	UTI	277 (3.6)	52 (1.3)	329 (2.7)	2.93	2.16–4.03	< 0.001
	DVT/PE	6 (0.1)	7 (0.2)	13 (0.1)	0.46	0.12–1.60	0.15
	Bedsores	47 (0.6)	18 (0.4)	65 (0.5)	1.40	0.80–2.57	0.21
	Sepsis	268 (3.5)	84 (2)	352 (2.9)	1.74	1.35–2.25	< 0.001

(a) NIHSS: National Institute of health stroke scale - NIHSS is a standardized assessment tool used to assess stroke severity based on neurological function. Score ranges from 0 to 42, with higher scores indicating greater stroke severity

(b) mRS: Modified Rankin Scale– mRS scale is used to evaluate disability and functional independence following stroke. Score ranges from 0 to 6, where 0 indicates no symptoms, 1–2 reflects slight disability, 3–5 indicates moderate to severe disability, and 6 represents death

IQR: Interquartile range, CI: Confidence interval, TIA: Transient ischemic attack, ICH: Intracerebral hemorrhage, CVST: Cerebral venous sinus thrombosis, UTI: Urinary tract infection, PE: Pulmonary embolism

**Table 3:** Association of emergency medical services utilization with management and outcomes in acute stroke care

Variables	Category	Relative odds of outcomes in patients utilizing EMS as mode of transportation					
		Unadjusted Odds Ratio	*p*-value	95% CI	Adjusted Odds Ratio	*p*-value	95% CI
DNT, (*n* = 783)		-19.3^+^	< 0.001	-26.5, -12.9	-19.0^	< 0.001	-26.2, -11.8
Thrombolysis		2.65	< 0.001	2.19–3.19	2.10	< 0.001	1.72–2.57
Thrombectomy		3.32	< 0.001	2.35–4.69	2.25	< 0.001	1.55–3.28
Disposition	*Home as Ref*						
	Rehab	2.71*	< 0.001	2.38–3.08	1.71	< 0.001	1.48–1.98
	Long term care	2.76	< 0.001	2.10–3.59	1.24	0.18	0.90–1.70
	Died in Hospital	2.17	< 0.001	1.64–2.87	0.77	0.16	0.54–1.11
	Other specialty	1.49	< 0.001	1.30–1.71	1.22	< 0.01	1.05–1.41
Length of stay, days		1.42^+^	< 0.001	1.08–1.76	-0.05^^^	0.74	-0.37, 0.26
Favorable outcome at discharge	mRS 0–2	0.42	< 0.001	0.38–0.46	0.60	< 0.001	0.54–0.67
Favorable outcome at 90 days	mRS 0–2	0.38	< 0.001	0.34–0.43	0.58	< 0.001	0.49–0.67

*P* values are derived from logistic regression models with adjusted odds ratio and adjusted mean differences

*Adjusted for age, sex, ethnicity, diagnosis, NIHSS score (stroke severity) at admission, diabetes, hypertension, dyslipidemia, deep vein thrombosis, prior coronary artery disease, atrial fibrillation, congestive heart failure, chronic kidney disease, and prior history of stroke and TIA

+Unadjusted mean difference

^Adjusted mean difference

## Discussion

This study is the first comprehensive analysis of a seven-year prospective stroke dataset to report nationwide utilization of EMS in stroke patients and associated patient characteristics and outcomes in the Middle Eastern region. The data indicates that two-thirds of the stroke patients utilized EMS for transportation to the hospital, which was significantly higher than regional [25, 26] and many international reports [6, 27–37]. Previous studies have reported that 22–65% of patients activate EMS at stroke onset [38–42], reflecting variation in behavioral response, likely influenced by previously reported factors such as public knowledge of the signs and symptoms of stroke, presence of a bystander at stroke onset, more obvious stroke symptoms and increasing stroke severity, age, race and ethnicity, and socioeconomic status [38, 43–45]. Although EMS utilization in stroke patients in our population was high, EMS utilization could be further increased by adopting strategies from countries with higher EMS awareness and utilization, as suggested by reports from Germany [46] and England [47]. 

In the study population, EMS use was more common among older patients (> 65 years), males, individuals of Asian ethnicity, and those with a history of hypertension, AF, CKD, and ICH patients. Furthermore, patients presenting within 1–6 h of stroke onset and with higher NIHSS scores upon admission were also more likely to utilize EMS. Previous research supports the observation that older patients [6, 35, 48, 49], those with ICH [6], and those with more severe stroke [6, 47, 48, 50] tend to have higher utilization of EMS, aligning with our findings. However, there is a lack of consensus in the literature about the correlation between the utilization of EMS and factors such as age, sex, ethnicity, diagnosis, and stroke severity [38, 39, 44]. For instance, contrary to our findings, a study in France reported no association between age, ethnicity, and diagnosis of ICH with EMS utilization [36]. Alternatively, several factors were associated with decreased utilization of EMS, including Arab ethnicity, particularly among the Qatari population, diagnosis of TIA and stroke mimics, low stroke severity (NIHSS < 5), stroke onset time of > 24 h, and initial symptoms perceived as non-urgent, such as numbness, ataxia, and headache aligning with previous research [30]. 

Consistent with previous reports, the findings indicate that a history of stroke or TIA was not associated with higher EMS utilization [6, 28, 39, 50]. One possible explanation for this could be the inadequate patient education on recognizing symptoms and lack of emphasis on the importance of promptly activating EMS when symptoms occur. Considering the recurrent nature of the condition, prioritizing the education of high-risk patients and their immediate relatives about the signs, symptoms, and risk factors of stroke and prompt EMS activation at onset, may affect timely care. Future educational programs should, therefore, aim to not only enhance stroke awareness and knowledge but also encourage urgent EMS activation at symptom onset within targeted population groups.

Beyond the specific symptoms that prompt a particular response, a higher stroke severity score is also associated with increased EMS utilization [29, 33]. The analysis indicated that patients are more likely to utilize EMS when stroke severity is high (NIHSS > 5) and initial symptoms include weakness, speech impairment, or loss of consciousness, consistent with findings from previous studies [51, 52]. This may be attributed primarily to the patient’s condition being perceived as serious and potentially life-threatening [29, 52, 53]. Moreover, the convenience of transporting stroke patients with more significant disabilities to the hospital under the close supervision of healthcare professionals and specialized pre-hospital support also plays a crucial role [29]. This implies that patients may activate EMS not necessarily due to recognizing stroke symptoms or suspecting a stroke, but because they perceive the severity of the symptoms as life-threatening and the need to seek immediate expert care [54]. Although the total number of deaths was higher in the EMS group, mortality in ischemic stroke and ICH remained statistically insignificant, likely reflecting the greater severity of illness and complications in EMS users rather than an independent effect of EMS utilization.

Our research findings align with Western reports, demonstrating a correlation between the utilization of EMS and expedited evaluations, reducing door-to-needle time [50, 55–57]. Consequently, patients who utilized EMS were more likely to receive thrombolysis and thrombectomy treatment compared to those who did not use EMS. Significantly, stroke patients who came to the hospital by ambulance services had a notable reduction in the time to receive r-tPA (*p* < 0.001). Previous work suggests that reduction of two minutes of time from onset to thrombolysis treatment is equivalent to gaining approximately two days of disability-adjusted life years (DALYs) [57, 58]. Improving EMS use via public health initiatives and focused educational programs to ensure timely medical interventions for stroke patients with subsequently improved thrombolysis rates thus could offer improved morbidity.

Trends in EMS utilization demonstrated a steady increase, from 55.6% in 2014 to 74.5% in 2020, reflecting increased awareness and improved integration of prehospital stroke care. However, EMS utilization was influenced by the COVID-19 pandemic, with varying effects observed across different regions [59–63]. In Qatar, EMS use significantly increased (*p* < 0.001), suggesting increased awareness of prehospital services, accentuated by pandemic-related restrictions on transportation. Whereas Canada, the USA, and South Korea, reported a decline in EMS use, potentially due to concerns regarding hospital safety and EMS system capacity [59, 61–63]. Germany, however, maintained stable EMS stroke referrals despite the pandemic [60]. Although EMS use in Qatar increased, thrombolysis (*p* < 0.001) and thrombectomy (*p* < 0.01) rates were lower in 2020, while DNT, although not statistically significant, increased during the pandemic period, aligning with global reports [64–66] possibly reflecting pandemic-related operational modifications, resource reallocation, and infection control measures. Additionally, an increasing trend in stroke mimics and their EMS utilization was observed. Since stroke mimics undergo the same EMS dispatch, ED evaluations, and neuroimaging as true stroke, they pose significant resource implications for the healthcare system. The peak in 2019, with nearly four in ten suspected stroke patients being non-stroke, highlights the need for a refined triage system. Optimizing stroke triage, increasing public education, and establishing standardized protocols may help mitigate unnecessary EMS activations, improve resource allocations, and ensure timely stroke care.

This study has several strengths that contribute to its robustness and relevance. Firstly, data were collected prospectively as part of a national stroke database for all suspected stroke patients, with accuracy further validated through independent verification of medical records, ensuring data reliability. Additionally, the analysis covers a seven-year period, providing a comprehensive understanding of trends in EMS utilization and associated factors. The large sample size and population diversity further enhance the generalizability of the findings, offering applicability to similar populations in other regions. However, the study has several limitations. As an observational study, research design does not allow for causal inference, limiting conclusions on cause-and-effect relationships. Furthermore, the lack of data on educational level and socioeconomic status restricted analysis of EMS utilization in relation to factors such as health literacy, income, and lifestyle. Additionally, patient location at stroke onset and pre-stroke health status were not assessed, factors which could influence EMS utilization, considering patients living closer to the hospital may self-present, and bedridden patients may choose EMS for convenience of transportation, respectively. Future research should aim to address these gaps to provide a more comprehensive understanding of EMS utilization in stroke care.

## Conclusion

EMS utilization rates in Qatar were higher than previous regional and international reports however there is potential for further improvement. Lower EMS use among younger patients, those of Arab ethnicity (particularly the Qatari population), and individuals with less overt symptoms highlight the need for targeted interventions. Furthermore, EMS utilization is associated with an increased likelihood of receiving treatment and shorter delays in care compared to other modes of transportation. Thus, public health initiatives should prioritize increasing awareness among underrepresented groups regarding the critical role of EMS transport in ensuring timely and effective care for stroke patients, while future research should address current gaps to optimize EMS utilization further and improve stroke outcomes.

## Electronic supplementary material

Below is the link to the electronic supplementary material.

Supplementary Material 1

## Data Availability

The data that support the findings of this study are available from Hamad Medical Corporation, but restrictions apply to the availability of these data, and so are not publicly available. Data is, however, available from the authors upon reasonable request and with permission of Hamad Medical Corporation.
