# Supplementary material for: Emergency medical services utilization in acute stroke in Qatar - an observational cohort study
Source: Int J Emerg Med. 2025 Mar 31;18:64. doi: 10.1186/s12245-025-00877-5 (PMC11960035; doi:10.1186/s12245-025-00877-5)
Supplement: Supplementary file 1 — Supplementary Material 1 [file 12245_2025_877_MOESM1_ESM.docx]

**Supplemental Table 1: Characteristics and demographic factors associated with the utilization of Emergency Medical Services in stroke patients at stroke onset**

| Variables | Category | Relative odds of EMS use by stroke patients (n=11,892) | | | | | |
| --- | --- | --- | --- | --- | --- | --- | --- |
|  |  | Unadjusted Odds Ratio | p-value | 95% CI | Adjusted Odds Ratio | p-value | 95% CI |
| Age, (years) |  | 1.00 | <0.05 | 1.00-1.01 |  |  |  |
| Sex | Male | 1.21 | <0.001 | 1.11-1.32 |  |  |  |
| Age Category, (years) | *<24 (Ref)* |  |  |  |  |  |  |
|  | 24-44 | 1.51 | <0.05 | 1.04-2.19 |  |  |  |
|  | 44-65 | 1.46 | <0.05 | 1.00-2.12 |  |  |  |
|  | 65-80 | 1.62 | <0.05 | 1.10-2.36 |  |  |  |
|  | >80 | 1.90 | <0.01 | 1.25-2.90 |  |  |  |
| Ethnicity | *Arabs* | 0.81 | <0.001 | 0.75-0.87 |  |  |  |
| *Arabs as Ref* | South Asian | 1.26 | <0.001 | 1.16-1.37 |  |  |  |
|  | Far Eastern | 1.12 | 0.09 | 0.98-1.28 |  |  |  |
|  | African | 1.23 | <0.05 | 1.02-1.49 |  |  |  |
|  | Caucasian | 1.22 | 0.11 | 0.96-1.56 |  |  |  |
|  | Other | 1.53 | 0.47 | 0.48-4.89 |  |  |  |
| Diagnosis | *IS as ref* |  |  |  |  |  |  |
|  | TIA | 0.64 | <0.001 | 0.56-0.72 |  |  |  |
|  | ICH | 2.43 | <0.001 | 2.09-2.84 |  |  |  |
|  | Mimic | 0.81 | <0.001 | 0.74-0.88 |  |  |  |
|  | CVST | 0.95 | 0.68 | 0.68-1.34 |  |  |  |
| BMI |  | 0.99 | 0.20 | 0.98-1.00 |  |  |  |
| HBA1C |  | 0.98 | 0.11 | 0.97-1.00 | 0.97 | <0.05 | 0.95-0.99 |
| Comorbids | Diabetes | 0.97 | 0.49 | 0.90-1.05 |  |  |  |
|  | Hypertension | 1.28 | <0.001 | 1.18-1.38 |  |  |  |
|  | Dyslipidemia | 0.96 | 0.26 | 0.88-1.03 |  |  |  |
|  | DVT | 0.92 | 0.81 | 0.47-1.78 |  |  |  |
|  | CAD | 1.05 | 0.47 | 0.93-1.18 |  |  |  |
|  | AF | 1.41 | <0.001 | 1.19-1.66 | 1.35 | <0.05 | 1.05-1.74 |
|  | CHF | 1.19 | 0.66 | 0.54-2.62 |  |  |  |
|  | CKD | 1.24 | <0.05 | 1.00-1.52 |  |  |  |
|  | smoking | 1.03 | 0.57 | 0.93-1.13 |  |  |  |
|  | Prior stroke | 1.02 | 0.70 | 0.91-1.15 |  |  |  |
|  | Prior TIA | 0.75 | 0.14 | 0.52-1.09 |  |  |  |
| NIHSS admission | 0 Ref |  |  |  |  |  |  |
|  | 1-4 | 1.14 | <0.01 | 1.05-1.25 |  |  |  |
|  | 5-15 | 2.41 | <0.001 | 2.15-2.70 | 1.86 | <0.001 | 1.52-2.27 |
|  | 16-20 | 4.14 | <0.001 | 3.17-5.42 | 2.88 | <0.001 | 1.91-4.34 |
|  | >20 | 3.98 | <0.001 | 3.08-5.15 | 3.33 | <0.001 | 1.93-5.74 |
| TOAST |  |  |  |  |  |  |  |
| *SVD as Ref* | LVD | 1.70 | <0.001 | 1.47-1.96 | 1.35 | <0.001 | 1.15-1.57 |
|  | CE | 1.63 | <0.001 | 1.39-1.91 | 1.33 | 0.001 | 1.12-1.59 |
|  | SDO | 1.36 | 0.001 | 1.13-1.65 |  |  |  |
|  | SUO | 1.74 | <0.01 | 1.23-2.45 | 1.69 | <0.01 | 1.16-2.48 |

IQR: Interquartile range, CI: Confidence interval, IS: Ischemic stroke, TIA: Transient ischemic attack, ICH: Intracerebral hemorrhage, CVST: Cerebral venous sinus thrombosis, BMI: Body mass index, HbA1C: Glycated hemoglobin, DVT: Deep vein thrombosis, CAD: Coronary artery disease, AF: Atrial fibrillation, CHF: Congestive heart failure, CKD: Chronic kidney disease, NIHSS: National Institute of health stroke scale and TOAST criteria: Trial of Org 10172 in acute stroke treatment criteria.

**Supplementary Table 2: Yearly trends in the use of Emergency Medical Services (EMS) by stroke mimics**

| Variable | Category | Use of Emergency Medical Services (EMS) by stroke mimics, n (%) | | Total mimics | Total suspected strokes | p-value |
| --- | --- | --- | --- | --- | --- | --- |
| Yearly trends in stroke mimics |  | Yes, n=2068 (%) | No, 1334 (%) | N=3402 (%) | N=11892 (%) |  |
|  | 2016 | 286 (28.3) | 194 (31.6) | 480 (14.1) | 1625 (13.6) | 0.16 |
|  | 2017 | 373 (29.4) | 219 (35.7) | 592 (17.4) | 1880 (15.8) | <0.01 |
|  | 2018 | 464 (30.9) | 336 (43.6) | 800 (23.5) | 2269 (19.1) | <0.001 |
|  | 2019 | 580 (33.5) | 424 (47.2) | 1004 (29.5) | 2631 (22.1) | <0.001 |
|  | 2020 | 365 (30.7) | 161 (39.6) | 526 (15.5) | 1594 (13.4) | <0.01 |

**Supplemental Table 3: Comparison of stroke onset time and presentation of stroke in the Emergency Department in EMS vs non-EMS group**

| Variables | Category | Use of Emergency Medical Services (EMS) | | Total | Odds Ratio | 95% CI | p-value |
| --- | --- | --- | --- | --- | --- | --- | --- |
|  |  | Yes, n=7734 (%) | No, n=4158 (%) | N=11892 (%) |  |  |  |
| Onset time | Less than 1 hour | 189 (2.4) | 127 (3.1) | 316 (2.6) | 0.79 | 0.62-1.00 | <0.05 |
|  | 1-3 hours | 2,259 (29.2) | 620 (14.9) | 2,879 (24.2) | 2.35 | 2.13-2.60 | <0.001 |
|  | 3-4.5 hours | 727 (9.4) | 264 (6.4) | 991 (8.3) | 1.53 | 1.32-1.77 | <0.001 |
|  | 4.5-6 hours | 388 (5) | 175 (4.2) | 563 (4.7) | 1.20 | 0.99-1.45 | <0.05 |
|  | 6-8 hours | 385 (4.9) | 195 (4.7) | 580 (4.8) | 1.06 | 0.88-1.27 | 0.48 |
|  | 8-12 hours | 292 (3.8) | 157 (3.8) | 449 (3.7) | 0.99 | 0.81-1.22 | 0.99 |
|  | 12-24 hours | 506 (6.5) | 299 (7.2) | 805 (6.7) | 0.90 | 0.77-1.05 | 0.17 |
|  | More than 24 hours | 2,089 (27) | 1863 (44.8) | 3,952 (33.2) | 0.45 | 0.42-0.49 | <0.001 |
|  | Unsure | 685 (8.8) | 389 (9.4) | 1,074 (9.0) | 0.94 | 0.82-1.07 | 0.36 |
|  | In hospital | 2 (0.03) | 0 (0) | 2 (0.01) |  |  | 0.29 |
|  | Wake up stroke | 212 (2.7) | 69 (1.7) | 281 (2.3) | 1.67 | 1.26-2.23 | <0.001 |
|  |  |  |  |  |  |  |  |
| Side effected | None | 2110 (27.3) | 1,214 (29.2) | 3,324 (27.9) | 0.91 | 0.83-0.98 | <0.05 |
|  | Left | 2,848 (36.8) | 1,576 (37.9) | 4,424 (37.2) | 0.95 | 0.88-1.03 | 0.24 |
|  | Right | 2,478 (32) | 1,217 (29.3) | 3,695 (31.1) | 1.13 | 1.04-1.23 | <0.01 |
|  | Both/Generalized | 298 (3.8) | 151 (3.6) | 449 (3.8) | 1.06 | 0.86-1.30 | 0.54 |
|  |  |  |  |  |  |  |  |
| Presenting symptom | Weakness | 5593 (72.3) | 2,831 (68.1) | 8424 (70.8) | 1.22 | 1.12-1.33 | <0.001 |
|  | Numbness | 355 (4.6) | 370 (8.9) | 725 (6.1) | 0.49 | 0.42-0.57 | <0.001 |
|  | Aphasia | 544 (7.0) | 151 (3.6) | 695 (5.8) | 2.00 | 1.66-2.42 | <0.001 |
|  | Neglect | 122 (1.6) | 34 (0.8) | 156 (1.3) | 1.94 | 1.31-2.93 | <0.001 |
|  | Gaze deviation | 81 (1.1) | 30 (0.7) | 111 (0.9) | 1.45 | 0.94-2.29 | 0.07 |
|  | Hemianopia | 125 (1.6) | 43 (1.0) | 168 (1.4) | 1.57 | 1.10-2.28 | <0.05 |
|  | Ataxia | 397 (5.1) | 272 (6.5) | 669 (5.6) | 0.77 | 0.65-0.90 | <0.01 |
|  | Diplopia | 235 (3.0) | 260 (6.3) | 495 (4.2) | 0.46 | 0.39-0.56 | <0.001 |
|  | Dysphagia | 112 (1.5) | 59 (1.4) | 171 (1.4) | 1.02 | 0.73-1.42 | 0.89 |
|  | Dysarthria | 1,964 (25.4) | 905 (21.8) | 2,869 (24.1) | 1.22 | 1.11-1.33 | <0.001 |
|  | Loss of consciousness | 610 (7.9) | 122 (2.9) | 732 (6.2) | 2.83 | 2.31-3.48 | <0.001 |
|  | Seizures | 177 (2.3) | 34 (0.8) | 211 (1.8) | 2.84 | 1.95-4.23 | <0.001 |
|  | Facial weakness | 951 (12.3) | 524 (12.6) | 1,475 (12.4) | 0.97 | 0.86-1.09 | 0.62 |
|  | Headache | 929 (12.1) | 623 (14.9) | 1,552 (13) | 0.77 | 0.69-0.86 | <0.001 |
|  | Dizziness | 1,602 (20.7) | 866 (20.8) | 2,468 (20.7) | 0.99 | 0.90-1.09 | 0.88 |

**Supplemental Table 4: Comparison of the utilization of EMS, patient characteristics, management and outcomes before (Pre-2020) and during the COVID-19 pandemic (2020)**

| Variable, n (%) | Category | Pre-2020 (2014-2019), n=10298 (86.5%) | 2020 (Jan-Sep), n=1594 (13.4%) | p-value |
| --- | --- | --- | --- | --- |
|  |  |  |  |  |
| EMS use, n (%) | Total | 6546 (63.5) | 1188 (74.5) | <0.001 |
|  | Ischemic stroke | 3253 (64.2) | 567 (76.5) | <0.001 |
|  | TIA | 568 (53.0) | 107 (69.0) | <0.001 |
|  | ICH | 951 (81.7) | 121 (88.3) | 0.054 |
|  | Mimic | 1703 (59.2) | 365 (69.4) | <0.001 |
|  | CVST | 71 (60.2) | 28 (80.0) | <0.05 |
| Age | Median, IQR | 52 (43-62) | 52 (43-63) | 0.06 |
| Male | N, (%) | 7787 (75.6) | 1172 (73.5) | 0.07 |
| Diagnosis, n (%) | Ischemic stroke | 5069 (49.2) | 741 (46.5) | <0.05 |
|  | TIA | 1071 (10.4) | 155 (9.7) | 0.41 |
|  | ICH | 1164 (11.3) | 137 (8.6) | <0.01 |
|  | Mimics | 2876 (27.9) | 526 (33) | <0.001 |
|  | CVST | 118 (1.2) | 35 (2.2) | <0.001 |
| NIHSS at admission | Median, IQR | 2 (0-5) | 2 (0-5) | 0.16 |
| Door-to-needle time | Median, IQR | 50 (32-72) | 62.5 (53-80.5) | 0.051 |
| Thrombolysis | N, (%) (IS) | 668 (13.2) | 57 (7.7) | <0.001 |
| Thrombectomy | N, (%) (IS) | 250 (4.9) | 17 (2.3) | <0.01 |
| Dead at discharge | n. (%) | 252 (2.5) | 25 (1.6) | <0.05 |
